# *Leishmania infantum* infection in European badgers (*Meles meles*) from northeastern Spain: a histopathological and immunohistochemical investigation

**DOI:** 10.1007/s00436-024-08369-w

**Published:** 2024-10-10

**Authors:** María Paz Peris, David Martínez-Durán, Patricia García, Chabier González, Mariano Morales, Juan Antonio Castillo, Juan José Badiola, Bernardino Moreno

**Affiliations:** 1 Department of Animal Pathology, Faculty of Veterinary Sciences, University of Zaragoza, Zaragossa, Spain; 2 Dept. of Agriculture, Livestock and Environment, Government of Aragón, Wildlife Rehabilitation Centre of La Alfranca (Zaragoza), La Alfranca S/N, Pastriz, Zaragossa, Spain; 3 Agrifood Institute of Aragón (IA2), Faculty of Veterinary Sciences, University of Zaragoza, Zaragossa, Spain

**Keywords:** *Leishmania* protozoa, Spain badger, Histopathological study, Immunohistochemistry

## Abstract

The European badger (*Meles meles*) is a common mustelid species known as a significant reservoir for various human and animal diseases. Studies investigating *Leishmania* infection in European badgers across Mediterranean regions have yielded inconsistent findings. In Spain, results are particularly controversial: some studies confirm the presence of *Leishmania* in badgers, while others do not. Our study aimed to conduct a retrospective histopathological and immunohistochemical analysis to detect *Leishmania* in tissues of nine European badgers from northeastern Spain, a region previously unevaluated for *Leishmania* infection in this species. Microscopic examination revealed lesions indicative of leishmaniosis in the lymph nodes and spleens of six badgers. In one of them, *Leishmania*-like structures were identified in multiple organs and confirmed via immunohistochemistry. Parasites were detected in the lymph nodes, spleen, adrenal glands, and pancreas. The parasite load was high in the adrenal glands, moderate in the lymph nodes and spleen, and low in the pancreas. No parasites were found in other examined organs. This finding represents a frequency of 11.11% (1/9) of *Leishmania* infection among the badgers we studied. Further investigation of wildlife and atypical reservoirs can enhance our understanding of the pathogenesis of this significant zoonotic disease.

## Introduction

Caused by protozoan parasites of the genus *Leishmania*, leishmaniosis is a vector-borne disease that represents a significant threat to human and animal health worldwide (WHO 2010). Infection is transmitted by the bite of female sandflies of the subfamily Phlebotominae, with *Phlebotomus perniciosus* acting as the most relevant biological vector in Europe (Maroli et al. 2013).

In most areas where *Leishmania* is endemic, infected and/or diseased dogs are known to be the main epidemiological reservoir, although other domestic and wild mammals have been found to be infected with *Leishmania* and have been proposed as secondary or alternative hosts (Millan et al., 2014; Tomassone et al. 2018). Recently, the Mustelidae family was found to carry *Leishmania* parasites in different regions of Mediterranean countries (Cardoso et al. 2021). Nevertheless, the ability to act as a competent reservoir, i.e., infecting sandflies when they are taking a blood meal, has only been confirmed in hares (*Lepus granatensis*), rabbits (*Oryctolagus cuniculus*), black rats (*Rattus rattus*), non-human-primates, maned wolves (*Chrysocyon brachyurus*), and bush dogs (*Speothos venaticus*) (Molina et al. 2012; Jiménez et al. 2014; Zanet et al. 2014; Mol et al. 2015; Rodrigues de Oliveira et al. 2019).

The European badger is a prevalent mustelid that is an important reservoir for human and animal diseases such as tuberculosis (Corner et al. 2012). Investigations of *Leishmania* infection in this species have been conducted in various Mediterranean countries with varying results (Del Río et al. 2014; Battisti et al. 2020; Magri et al. 2022). In Spain, results are inconclusive. Some authors have demonstrated the presence of *Leishmania* in European badgers (Del Río et al. 2014), while others have not found any traces (Risueño et al. 2018; Oleaga et al. 2018; Alcover et al. 2020).

Our objective was to carry out a retrospective histopathological and immunohistochemical study to detect *Leishmania* in the tissues of several European badgers in northeast Spain, an area where *Leishmania* in European badgers has not been previously evaluated.

## Materials and methods

### Animals and sampling

Nine European badgers received at the Wildlife Rehabilitation Center of La Alfranca (Aragón, Spain) during 2019 were included in this study. The causes of admission were variable. Postmortem examination was carried out on all of them, and samples were taken for histopathological evaluation. In all of these, at least one classic target organ for the detection of canine leishmaniosis was available: lymph nodes, spleen, and/or liver. The date of admission of each European badger, the sex, the age, the causes of death, and the tissues we analyzed are described in Table 1. The pathological evaluation was carried out by two pathologists (JJB and BM).

**Table 1:** Data and samples received by the Wild Species Recovery Center of La Alfranca (Zaragoza, Spain)

Identification	Sex	Age	Month	Death cause	Tissues sampled
Badger 1	Male	Old	January	Emaciation	Lymph node, spleen, liver, lung, heart, pancreas, kidney, small intestine, adrenal gland
Badger 2	Female	Young	February	Pneumonia	Lymph node, spleen, liver, lung, heart and kidney
Badger 3	Male	Adult	February	Trauma	Lymph node, spleen, liver, lung, small intestine
Badger 4	Female	Old	March	Trauma	Lymph node, spleen, liver, lung
Badger 5	Male	Old	March	Emaciation	Lymph node, spleen, liver, lung, kidney, small intestine
Badger 6	Female	Adult	March	Mycosis	Lymph node, spleen, liver, lung, pancreas, kidney, small intestine
Badger 7	Male	Adult	August	Trauma	Liver and lung
Badger 8	Female	Adult	October	Pneumonia	Lymph node, spleen, liver, lung, kidney
Badger 9	Male	Young	December	Septicemia	Lymph node, spleen, liver, lung, heart, pancreas, kidney, small intestine

^*^The age was estimated based on the appearance of the teeth

### Sample processing and histopathological evaluation

Samples were fixed in 10% neutral-buffered formalin for 48 h at room temperature. Then, they were embedded in paraffin wax and cut at 4 μm. Sections were stained with hematoxylin and eosin (H&E) and examined using light microscopy. In addition, special stains such as Ziehl–Neelsen for mycobacteria and Periodic Acid Schiff (PAS) for fungi were also carried out.

We particularly looked for characteristic lesions of canine leishmaniosis, such as granulomatous and/or lymphoplasmacytic inflammation. We assessed lesion intensity as follows: − / + , isolated foci; + , mild; +  + , moderate; +  +  + , intense; − , absent.

### Immunohistochemical evaluation

Immunohistochemical (IHC) evaluation was carried out as described in an earlier publication (Peris et al. 2022). Briefly, IHC was performed in an Autostainer Plus Staining System (Dako Cytomation, Denmark), with a specific rabbit antiserum raised against *L. infantum* (donated by Dr. Ricardo Molina, Servicio de Inmunología, Instituto de Salud Carlos III, Madrid, Spain) as primary antibody. The antiserum was diluted at 1:6000 and incubated for 1 h. Antigen unmasking was performed on a Dako PT Link module at 96 °C for 20 min. Positive and negative controls were included in all immunohistochemistry runs. As a positive control, we used a lymph node stemming from a natural case of canine leishmaniosis in which *Leishmania* antigens had been previously found. For negative controls, PBS was used instead of the primary antibody.

We assessed the presence of parasites by comparison with the positive control using the following scale: + , low; +  + , moderate; +  +  + , high; − , absence.

## Results

**Fig. 1:**
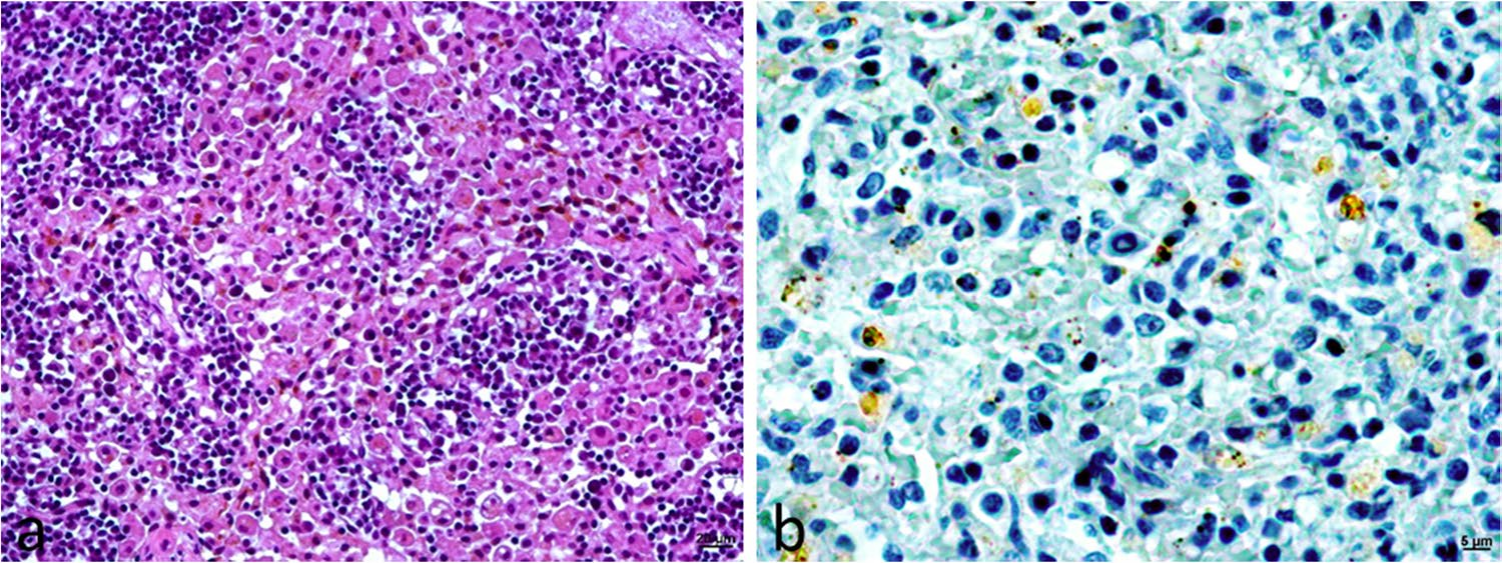
Lymph node of European badger 1. **a** Moderate infiltration of macrophages mainly located in sinusal areas of the cortex. HE, 200 × . **b** Amastigote forms located within macrophages and between them. IHC, 630 ×

**Fig. 2:**
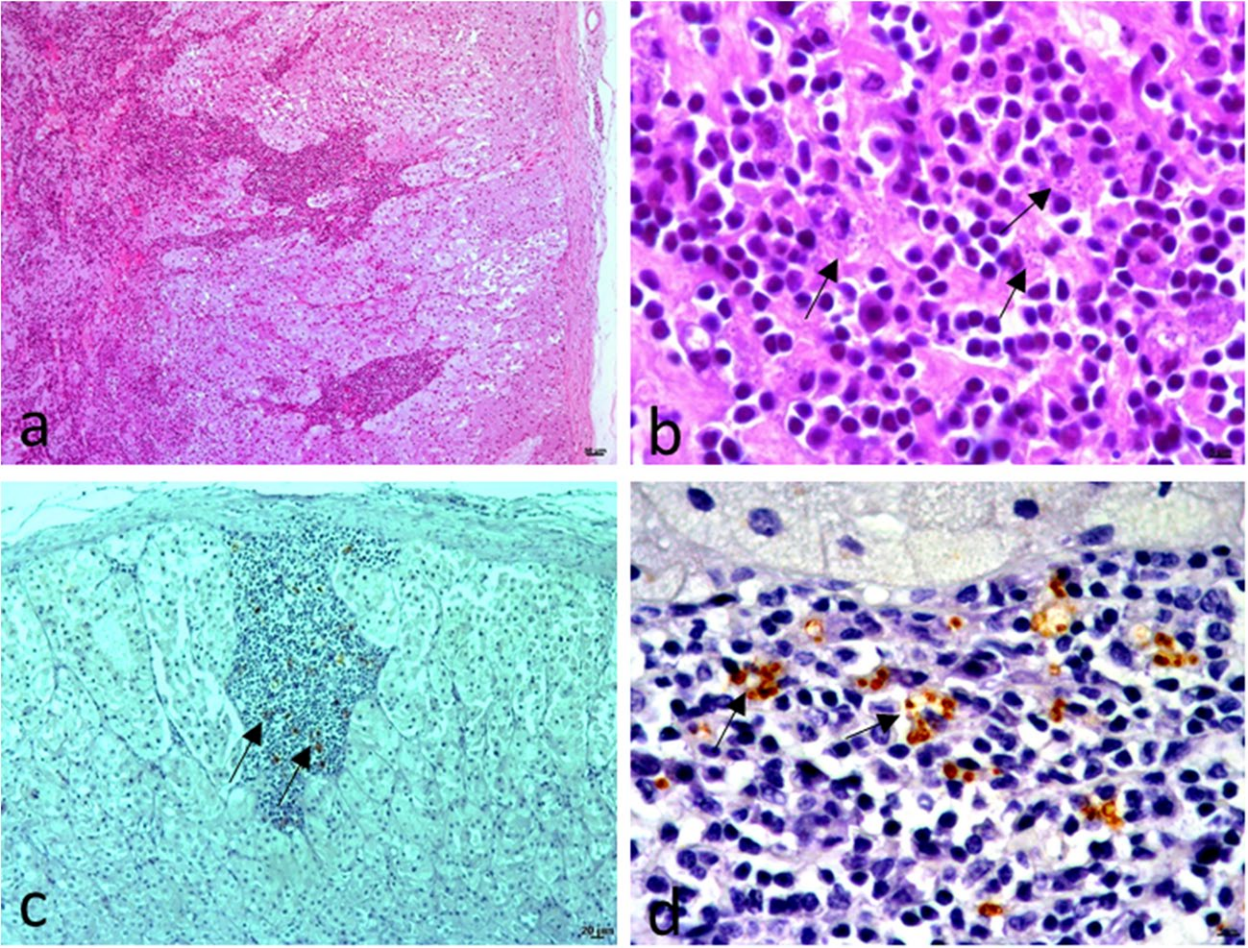
Adrenal gland of European badger 1. **a** Moderate multifocal adrenalitis composed mainly of macrophages and lymphocytes. HE, 200 × . **b** Detail of inflammation with structures compatible with protozoa inside macrophages (arrows). HE, 630 × . **c** and **d** Parasites are observed as brown structures associated with the inflammatory reaction (arrows). IHC. **c** 100 × . **d** 400 ×

The recovery center did not report enlargement of peripheral lymph nodes or skin lesions such as papular and/or ulcerative dermatitis in any of the badgers. Microscopic examination (Table 2) revealed lesions compatible with leishmaniosis in the lymph nodes and spleen of six animals. They were characterized by mild to moderate multifocal macrophage infiltration, some of them forming granulomas (Fig. 1a). Lesions were also observed in the adrenal gland of a badger (Fig. 2a). In this badger, structures compatible with *Leishmania* were observed in several organs, which were later confirmed by immunohistochemistry (Table 3). Parasites were observed in the lymph nodes (Fig. 1b), spleen, adrenal glands (Fig. 2b, c, d), and pancreas. The *Leishmania* burden was high in the adrenal glands, moderate in the lymph nodes and spleen, and low in the pancreas. No parasites were found in other organs.

**Table 2:** Histopathological findings. Assessment of inflammatory lesion compatible with *Leishmania* (lymphoplasmacytic and/or granulomatous inflammation) and lesional grade (− / + , isolated foci; + , mild; +  + , moderate; +  +  + , intense; − , absence; *NA*, sample not available)

Number	Lymph node	Spleen	Liver	Lung	Heart	Pancreas	Kidney	Small intestine	Adrenal gland
Badger 1	+	+	− / +	−	−	−	− / +	NA	+ +
Badger 2	−	−	−	−	−	NA	−	NA	NA
Badger 3	−	+	−	−	NA	NA	NA	−	NA
Badger 4	+ +	−	−	+ +	NA	NA	NA	NA	NA
Badger 5	−	−	−	−	NA	NA	−	−	NA
Badger 6	+ +	−	−	−	NA	−	−	−	NA
Badger 7	NA	NA	−	−	NA	NA	NA	NA	NA
Badger 8	+	+	−	−	NA	NA	−	NA	NA
Badger 9	+	−	−	−	−	−	−	−	NA

**Table 3:** Immunohistochemical results. Parasite density (+ , low; +  + , moderate; +  +  + , high; − , absence; *NA*, sample not available)

Number	Lymph node	Spleen	Liver	Lung	Heart	Pancreas	Kidney	Small intestine	Adrenal gland
Badger 1	+	+	−	−	−	+	−	NA	+ + +
Badger 2	−	−	−	−	−	NA	−	NA	NA
Badger 3	−	−	−	−	NA	NA	NA	−	NA
Badger 4	−	−	−	−	NA	NA	NA	NA	NA
Badger 5	−	−	−	−	NA	NA	−	−	NA
Badger 6	−	−	−	−	NA	−	−	−	NA
Badger 7	NA	NA	−	−	NA	NA	NA	NA	NA
Badger 8	−	−	−	−	NA	NA	−	NA	NA
Badger 9	−	−	−	−	−	NA	−	−	NA

Immunohistochemistry did not reveal parasitic structures in the remaining badgers. Ziehl–Neelsen staining did not reveal acid-alcohol-resistant pathogens in any of the samples, nor did PAS staining reveal any fungal structures. Most granulomas were apparently associated with foreign material.

## Discussion

In Mediterranean countries, dogs are considered the main domestic reservoir of *Leishmania* in urban areas. However, the role of wildlife in the epidemiology of *Leishmania* is being increasingly discussed, as *Leishmania* has been detected in numerous wild species, including canids, felids, mustelids, lagomorphs, and rodents (Millán et al. 2014; Tomassone et al. 2018; Abbate et al. 2019; Cardoso et al. 2021). Nevertheless, most studies have focused on wild canids due to their similarity with dogs while mostly ignoring other species, such as mustelids and rodents. Moreover, most studies have used molecular or serological methods (Magri et al. 2022; Battisti et al. 2020; Taddei et al. 2022; Abbate et al. 2019). Molecular methods such as PCR offer high sensitivity; however, the presence of DNA of a particular parasite may not always indicate active infection (Brinsko 2004). In addition, all those studies were carried out on target organs, such as the spleen, the lymph nodes, or skin. Few studies, if any, have described pathologic findings associated with parasite distribution (Gomes et al., 2020).

European badgers are mustelids in which *Leishmania* has been increasingly reported in various Mediterranean countries (Cardoso et al. 2021). In Spain, Del Rio et al. (2014) reported a 26.4% prevalence of *Leishmania* in liver and spleen samples from 14 European badgers in the northern area, while Azami-Conesa et al. (2023) found a prevalence of 35.71% in liver and skin samples from 14 European badgers in regions including Madrid, Castilla-La Mancha, Castilla y León, and the Valencian Community. However, further studies in Spain, including one in the southeast (Risueño et al. 2018), one in the north (Oleaga et al. 2018), and one in the Mediterranean regions (Alcover et al. 2020), did not yield any positive results.

Molecular techniques have also revealed the presence of *Leishmania* DNA in other mustelids, although few have actually featured lesions or symptoms compatible with the infection. A Eurasian otter (*Lutra lutra*) housed at a wildlife park in Murcia (Cantos-Barreda et al. 2020) and a domesticated ferret (*Mustela putorius furo*) in Valencia displayed characteristic cutaneous lesions as a clinical sign of *Leishmania* (Giner et al. 2020). Unfortunately, no samples for molecular or serological analyses could be taken in this study due to logistical reasons.

To our knowledge, this study marks the first confirmation of the presence of *Leishmania* in European badgers in Aragón, with a frequency of 11.11% in nine sampled European badgers. To obtain a more robust prevalence estimate in Aragón, further sampling will be necessary.

The organ targets of *Leishmania* infection are mainly the lymph nodes and the spleen (Koutinas and Koutinas 2014). In our study, *Leishmania* was indeed detected in both of those organs. Interestingly, we also noted the presence of *Leishmania* in atypical organs such as the pancreas and adrenal gland, with particular severity in the latter. Parasite infection of the adrenal gland has been sporadically reported, with one description in a maned wolf (*Chrysocyon brachyurus*) and a small number of dogs (Momo et al. 2014; Carvalho et al. 2015). Despite limited adrenal samples, the consistency of positivity in lymph nodes and spleen reinforces the importance of these organs for the diagnosis of leishmaniosis in European badgers (Portús et al. 2002). Our study’s results suggest that adrenal glands should be routinely screened for the presence of *Leishmania* parasites.

Lesions induced by *Leishmania* infection in dogs are characterized by granulomatous and lymphoplasmacytic inflammation (Koutinas and Koutinas 2014), although gross lesions may sometimes be atypical, leading to misleading diagnoses (Peris et al. 2022). Descriptions of the disease’s effects on other species are less clearly defined. Granulomatous lesions can be observed in other conditions, such as tuberculosis, fungal infections, foreign material, or atypical pathogens (Gavier-Widen et al. 2001; Canfield et al. 2002; Moreno et al. 2015).

In the UK, European badgers have been shown to be important reservoirs of tuberculosis (Atkins and Robinson 2013). However, this does not seem to be the case in Spain, where wild boar and deer are the primary reservoirs (Gortázar et al., 2012; Santos et al. 2022). Studies on European badgers have found a certain degree of prevalence of tuberculosis in northern Spain, where cattle are abundant and tuberculosis is prominent; in Aragón, however, tuberculosis is sporadic (Balseiro et al. 2013; Acevedo et al. 2019).

No gross lesions were observed in the badgers in our study; however, microscopy revealed small granulomas and groups of epitheliod macrophages in the lymph nodes. Those lesions are similar to tuberculosis lesions observed in carnivores, where epithelioid granulomas predominate (Canfield et al. 2002). Granulomatous lesions should always be evaluated with specific stains, such as Ziehl–Neelsen for mycobacteria or PAS for fungi, or with the aid of more sensitive techniques, such as immunohistochemistry, for several pathogens. In the present study, Ziehl–Neelsen staining did not reveal acid-alcohol-resistant pathogens in any of the samples, nor did routine stains reveal any fungal structures.

Sandflies play a crucial role in disease transmission, as their ability to act as reservoirs for *Leishmania* depends on their capacity to infect other hosts (Gradoni et al., 2013; Pozio et al. 1985). The sandfly is known to nest in burrows such as those created by rabbits and hares, a habitat that has been implicated in the epidemiology of leishmaniosis outbreaks, as recently reported in Madrid (Molina et al. 2012; Jiménez et al., 2014; González et al. 2017; González et al. 2021). Given that European badgers also make burrows (Virgos and Casanovas 1999), it is plausible to assume that sandflies may also inhabit them and easily infect the badgers who made them.

*Leishmania* infection may depend on seasonal vector activity (Risueño et al. 2018). The infected European badger featured in the present study was found in January, a month with low vector activity. In Aragón, located in the northeast of Spain, canine leishmaniosis is transmitted by two sandfly species, *P. ariasi* and *P. perniciosus*, which are active during the warmer months of the year, typically from May to October (Lucientes-Curdi et al. 1991). Interestingly, the duration of the sandfly season does not significantly affect the prevalence of parasite infection or seroprevalence in dogs (Fernández-Bellon et al., 2018). This makes the transmission of *Leishmania* infections via European badgers all the more plausible.

In conclusion, our study has demonstrated the presence of a disseminated *Leishmania* infection in a European badger encouraging further in-depth study of wildlife and atypical locations to enhance our understanding of the pathogenesis of this important zoonosis.

## Data Availability

No datasets were generated or analyzed during the current study.
